# Activity of Secondary Metabolites from Marine-Derived *Trichoderma longibrachiatum* ST-27 Against *Vibrio harveyi*

**DOI:** 10.3390/ijms27177714

**Published:** 2026-08-28

**Authors:** Youying Chen, Gengtao Chen, Cheng-Yang Hsieh, Zhiwen Wu, Xu Tang, Po-Wei Tsai, Yung-Husan Chen

**Affiliations:** 1 Xiamen Key Laboratory of Natural Products Resources of Marine Medicine, Xiamen Medical College, Xiamen 361023, China; chenyouying@xmmc.edu.cn (Y.C.); 2250320412@fjmu.edu.cn (G.C.); 2 Fujian Provincial University Marine Biomedical Resources Engineering Research Center, Xiamen Medical College, Xiamen 361023, China; 3 Department of Food Science, National Taiwan Ocean University, Keelung 202, Taiwan; d339108001@tmu.edu.tw; 4 Department of Chemical and Materials Engineering, National I-Lan University, Yilan 260, Taiwan; 5 Central Region Campus, Industrial Technology Research Institute, Nantou 540, Taiwan; 6 Engineering Research Center of Marine Biological Resource Comprehensive Utilization, Third Institute of Oceanography, State Oceanic Administration, Xiamen 361005, China; wuzhiwen@tio.org.cn (Z.W.); tangxu@tio.org.cn (X.T.)

**Keywords:** antibacterial activity, aquaculture, *Marine fungi*, peptaibols, secondary metabolites, *Trichoderma longibrachiatum*

## Abstract

Aquaculture and environmental challenges continue to constrain the industry’s sustainable development. The misuse of antimicrobial agents has accelerated the dissemination of resistance genes, posing a serious threat to both animal husbandry and human health. Thus, the discovery of novel antibiotic alternatives is of great significance for sustainable aquaculture and public health protection. *Marine fungi* have recently attracted considerable attention in natural product research due to their ability to produce structurally diverse and bioactive secondary metabolites under unique marine conditions. In this study, the marine-derived strain *Trichoderma longibrachiatum* ST-27 was subjected to large-scale fermentation. The crude extract (ST-27-3) was fractionated into seven components using gel permeation chromatography, followed by further purification with silica gel column chromatography and semi-preparative high-performance liquid chromatography (HPLC). Two compounds were isolated, among which two were identified as pentadecaibin IV (**1**) and trichorzianine 1938 (**2**) by nuclear magnetic resonance (NMR) and high-resolution mass spectrometry (HRMS) analyses. Their antibacterial activities were subsequently evaluated. Pentadecaibin IV and trichorzianine 1938 are linear small-molecule polypeptides. Antibacterial assays showed that trichorzianine 1938 exhibited concentration-dependent inhibitory activity against *Vibrio harveyi* VH306, with the highest inhibition observed at 300 µg/mL. These findings demonstrate that *T. longibrachiatum* ST-27 is a rich source of peptaibol-type metabolites with antibacterial, antifungal, insecticidal, and cytotoxic properties. The results highlight the potential of marine-derived peptaibols as promising lead compounds for the development of novel antimicrobial agents and provide a theoretical basis for their application in aquaculture disease control.

## 1. Introduction

Unique conditions, including high hydrostatic pressure, elevated salinity, low dissolved oxygen, steep temperature gradients, limited light penetration, and nutrient scarcity, characterize marine ecosystems. Within this complex ecological context, marine microorganisms form intricate symbiotic networks with their hosts. These unique physiological and biochemical mechanisms enable marine microbes to synthesize structurally diverse and biologically active secondary metabolites with novel molecular scaffolds [1,2,3].

The genus *Trichoderma* belongs to the family Hypocreaceae, order Hypocreales, class Sordariomycetes, and phylum Ascomycota [4]. Species of this genus are widely distributed and have attracted considerable attention because of their ecological importance and their ability to produce structurally diverse bioactive secondary metabolites; they are widespread fungi that have attracted increasing attention due to their significant potential in biotechnology. In recent years, secondary metabolites from *Trichoderma* species have drawn substantial interest owing to their pronounced biological activities and broad research significance. The remarkable chemical diversity of *Trichoderma* metabolites forms the foundation for their promising applications in biological control and natural product discovery [4].

Many *Trichoderma* species produce bioactive linear non-ribosomal peptides (NRPs), commonly known as peptaibols. These peptides typically have molecular weights ranging from 500 to 2000 Da, comprising 5–20 amino acid residues, an N-terminal acetyl group, and a C-terminal amino alcohol. Peptaibols represent one of the most important subclasses of fungal peptide natural products, with numerous sequences reported and cataloged in specialized databases [5].

Peptaibols have attracted research interest due to their diverse biological activities and potential as alternative antibiotics or novel therapeutic agents [6]. They exhibit broad-spectrum activities, including antibacterial effects against Gram-positive bacteria and dormant Mycobacterium spp., as well as antifungal and antiviral properties, particularly against tobacco mosaic virus. Mechanistically, peptaibols form pores or voltage-dependent ion channels in microbial membranes, thereby increasing membrane permeability and disrupting cellular integrity.

For example, Zhao et al. [7] isolated a novel heterodipeptide, trichodermamide G, from *Trichoderma harzianum* D13 associated with mangrove plant roots in Hainan, China. Structural elucidation revealed a unique sulfur-bridged diketopiperazine moiety, and the compound showed antifungal activity against *Magnaporthe oryzae* and *Valsa mali*, with MIC values of 17.4 and 16.7 μmol/L, respectively.

Similarly, Sun et al. [8] obtained a new cyclotetrapeptide, trichoderide A, from *T. reesei* YZ48-08 isolated from marine sediment in Lianyungang, China, which significantly inhibited the proliferation of *human melanoma* A375-S2 cells (IC_50_ = 18.5 μg/mL).

More recently, Tang et al. [9] reported seven novel 18-residue peptaibols, neoatroviridins E–K, from *Trichoderma* MT594385.1, and confirmed their absolute configurations using NMR and Marfe’s analysis. Antibacterial evaluation showed moderate inhibitory activity against *Staphylococcus aureus*, with MIC values ranging from 8 to 32 μg/mL. Neoatroviridins I, which features Iva residues at the R1 (C-5), R2 (C-7), and R3 (C-11) positions, demonstrated the highest antibacterial efficacy against Staphylococcus aureus, indicating a structure–activity relationship.

With the continuous advancement of aquaculture technologies, this sector has become a vital source of high-quality protein, essential polyunsaturated fatty acids, and micronutrients for humans [10]. However, the rapid intensification of aquaculture practices has led to frequent outbreaks of infectious diseases, compounded by deteriorating environmental conditions. Moreover, the misuse of antibiotics has facilitated the dissemination of resistance genes, impeding disease control in animal husbandry and posing risks of antimicrobial resistance transmission to humans. In response, the Chinese Ministry of Agriculture has implemented stringent regulations restricting the use of antibiotics in aquaculture, reducing their application as feed additives for disease prevention and growth promotion. Consequently, the development of efficient antibiotic alternatives is urgently needed to promote sustainable aquaculture and safeguard public health. Antimicrobial peptides (AMPs), as crucial components of innate immunity, have emerged as promising candidates due to their broad-spectrum antibacterial, antiviral, antifungal, antiparasitic, and antitumor activities, as well as their ability to enhance host immunity and growth [11].

For example, in 2020, China’s aquaculture production reached 52.24 million tons, accounting for 79.8% of total aquatic output. However, high-density farming has exacerbated disease outbreaks, causing major economic losses [12,13]. AMPs provide a potential solution by effectively inhibiting pathogenic microorganisms in aquaculture environments, thereby offering a sustainable alternative to conventional antibiotics. Recent studies have demonstrated the efficacy of various AMPs in aquaculture. Frog-derived caerin 1.1, fish-derived dicentracin, and zebrafish-derived NK-lysin peptide (NKLP) exhibit potent antimicrobial and antiviral activities in vitro [14]. NKL-24, another AMP derived from zebrafish, inhibits *Vibrio parahaemolyticus* by disrupting cell membranes [15]. Furthermore, Hu et al. [16] reported that grouper-derived AMP EmPis-1 effectively suppressed antibiotic-resistant bacteria at sub-10 μmol/L concentrations by compromising cell membrane integrity, demonstrating activity against *Escherichia coli*, *Staphylococcus aureus*, and *Vibrio parahaemolyticus*.

Among aquaculture pathogens, vibriosis, caused by *Vibrio* spp., is particularly devastating due to its rapid spread, broad host range (shrimp, fish, shellfish, and crustaceans), and high pathogenicity, resulting in severe economic losses worldwide [17]. Thus, exploring AMPs and peptaibol compounds as alternatives to antibiotics represents an important strategy for controlling *Vibrio* infections in aquaculture.

The present study investigates the secondary metabolites of the marine-derived *T. longibrachiatum* ST-27, focusing on their antibacterial activity against *V. harveyi*. The findings aim to provide additional experimental evidence for the potential application of marine-derived beneficial fungi in aquaculture disease management.

## 2. Results

### 2.1. Purity Analysis of Compounds

The target compounds were efficiently isolated and enriched using semi-preparative high-performance liquid chromatography (HPLC), yielding the following fractions: ST-27-4-3-1-3 (21.3 mg) and ST-27-4-3-1-6 (19.6 mg). Analytical HPLC was used to analyze the purity of these fractions. Before analysis, the samples were diluted and transferred into HPLC vials. Chromatographic separation was performed on a Welch Ultimate XB-C18 reversed-phase column (4.6 × 250 mm, 5 μm) using an isocratic elution program with a mobile phase consisting of acetonitrile and water (containing 0.1% trifluoroacetic acid). The analysis was conducted under the following conditions: a constant flow rate of 0.8 mL/min, multi-wavelength detection at 210, 230, 254, 280, and 310 nm, and an injection volume of 30 μL. Throughout the 60-min analysis, solvent B (acetonitrile) was maintained at 60%.

HPLC analysis confirmed that both ST-27-4-3-1-3 and ST-27-4-3-1-6 were highly pure and were reserved for subsequent structural characterization by 1H and ^13^C NMR spectroscopy.

After isolation and purification, the compounds were dissolved in deuterated dimethyl sulfoxide (DMSO-*d*_6_) and transferred to NMR tubes for structural analysis. The structures were elucidated based on comprehensive spectroscopic data, including ^1^H NMR,^13^C NMR, and high-resolution mass spectrometry (HRMS).

### 2.2. Identification of Compounds

#### 2.2.1. Compound **1**

Compound **1** was obtained as a pale yellow powder. Its molecular formula was determined to be C_70_H_117_N_17_O_18_ based on high-resolution electrospray ionization mass spectrometry (HR-ESI-MS), which exhibited a protonated molecular ion [M + H]^+^ at *m*/*z* 1484.83. The corresponding NMR and HR-ESI-MS spectra are provided in Figures S1–S3. NMR spectra displayed 17 exchangeable amide protons (*δ*_H_ 6.07–8.05 ppm) (Table 1), characteristic of Aib (α-aminoisobutyric acid) residues, glutamine side chains, glycine, alanine, and leucine residues. Aromatic signals at *δ*_H_ 6.20–6.41 ppm corresponded to monosubstituted phenyl groups.

**Figure 1:**
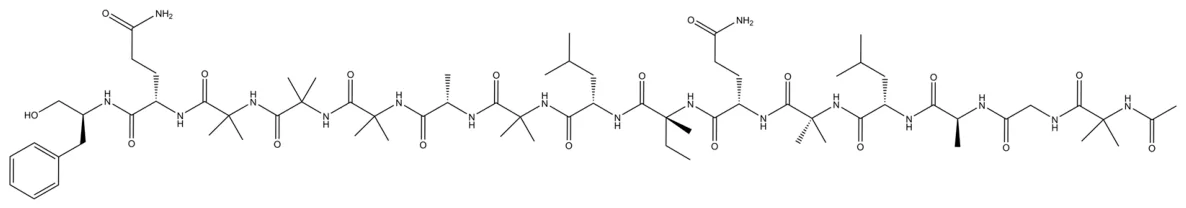
Compound **1** (Pentadecaibin IV).

**Figure 2:**
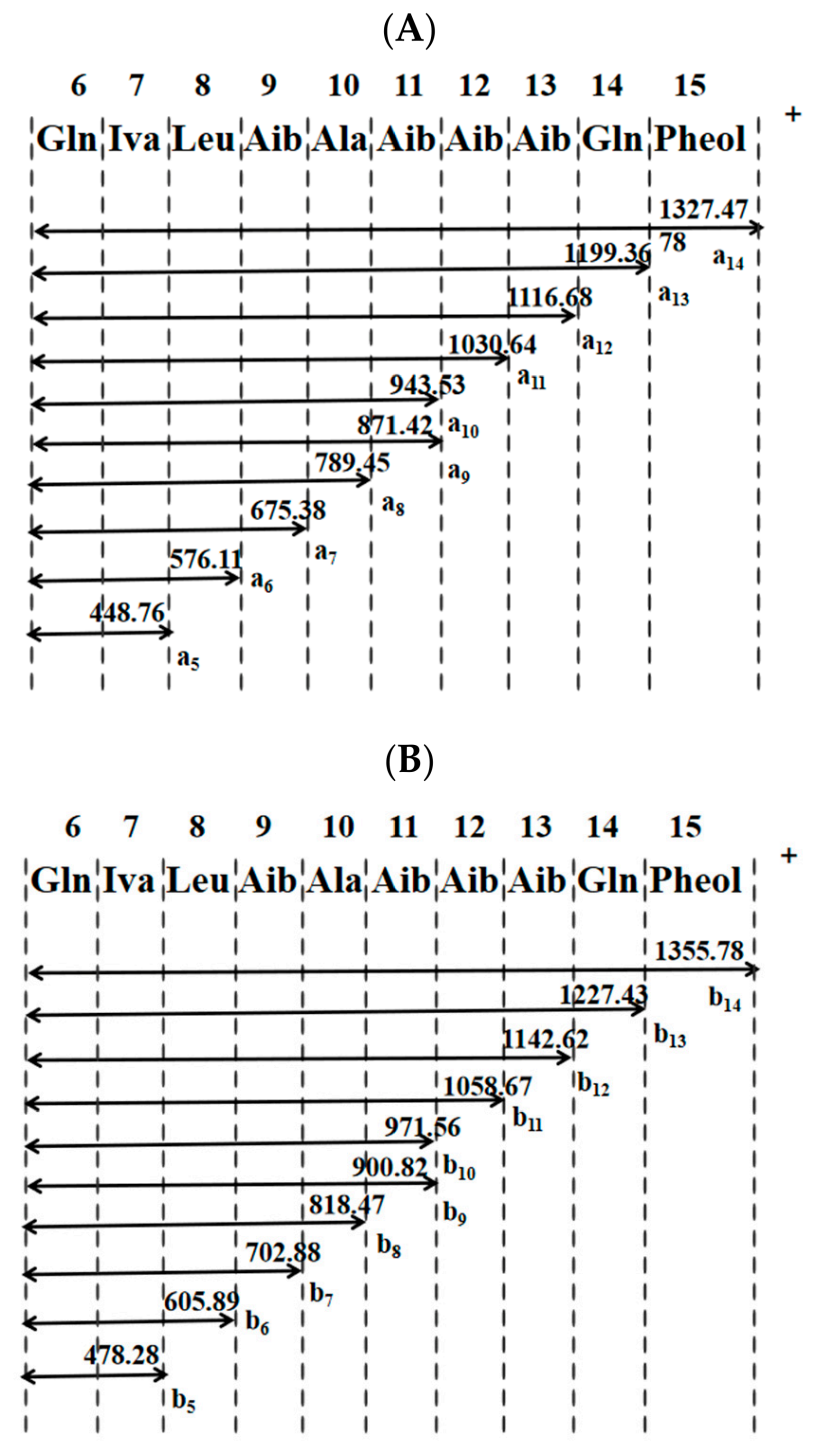

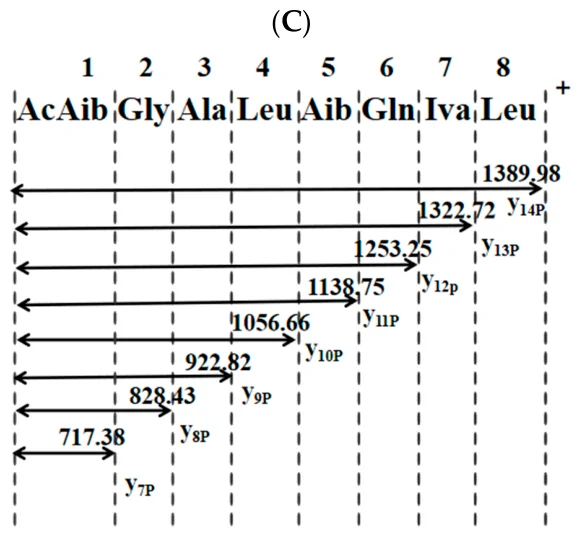
ESI-MS^2^ fragmentation pattern of pentadecaibin IV (Compound **1**): (**A**) a-type fragment ion series; (**B**) b-type fragment ion series; and (**C**) y-type fragment ion series, supporting the sequence assignment of Compound **1**.

ESI-MS^2^ fragmentation confirmed the amino acid sequence: Ac-Aib^1^-Gly^2^-Ala^3^-Leu^4^-Aib^5^-Gln^6^-Iva^7^-Leu^8^-Aib^9^-Ala^10^-Aib^11^-Aib^12^-Aib^13^-Gln^14^-Pheol^15^ (Figure 1 and Figure 2).

This was consistent with previously reported pentadecaibin IV [18].

#### 2.2.2. Compound **2**

Compound **2** was obtained as a pale-yellow powder. Its molecular formula was determined to be C_91_H_151_N_21_O_25_ based on high-resolution electrospray ionization mass spectrometry (HR-ESI-MS), which exhibited a protonated molecular ion [M + H]^+^ at *m*/*z* 1939.11. The corresponding NMR and HR-ESI-MS spectra are provided in Figures S4–S6. NMR spectra exhibited multiple Aib residues, including eight amide proton signals (*δ*_H_ 7.47–8.56 ppm) and α-carbon signals (*δ*_C_ 55.8–56.3 ppm) (Table 2). Characteristic methyl signals were observed at δH 1.30–1.49 ppm.

**Figure 3:**
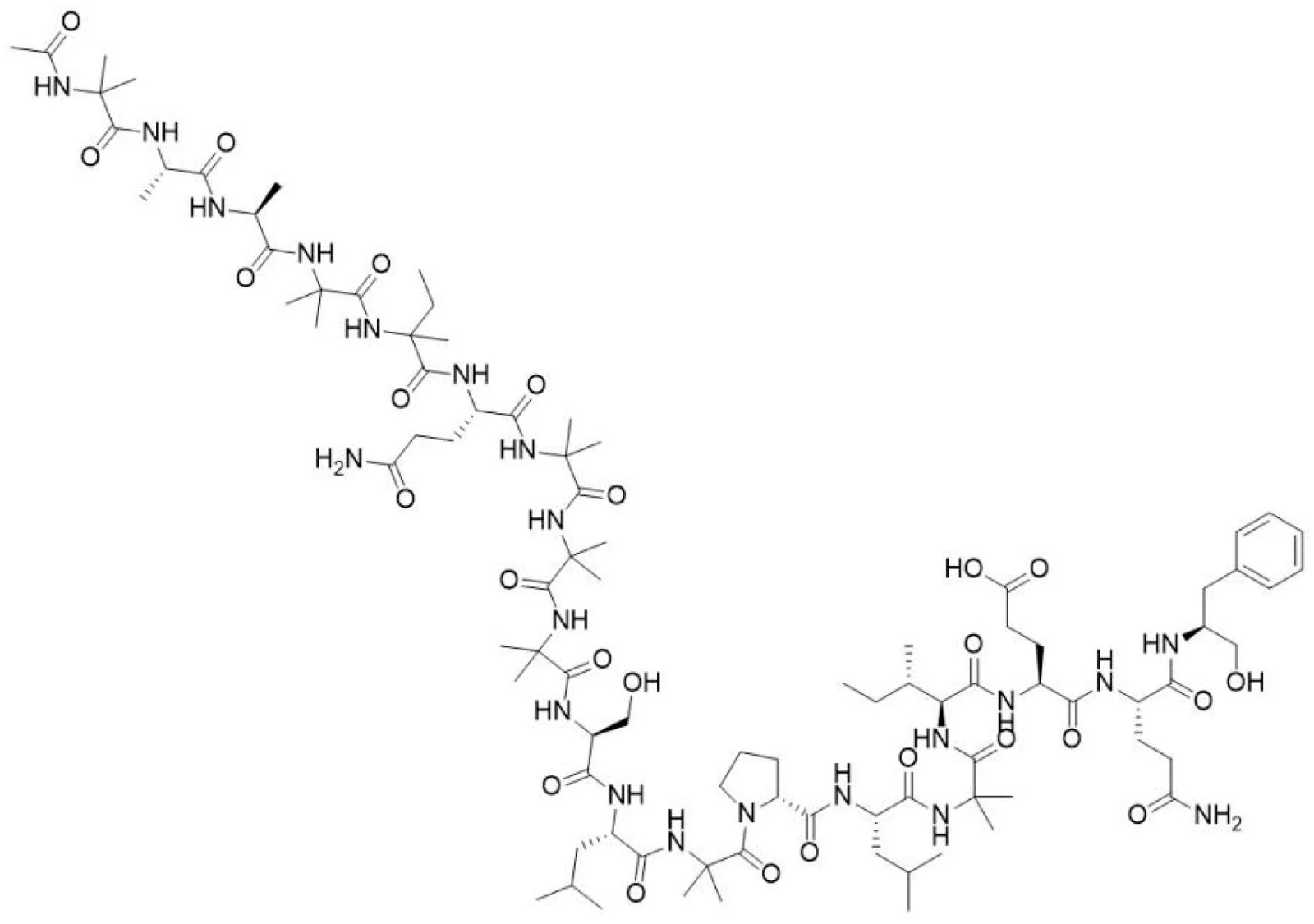
Compound **2** (Trichorzianine 1938).

**Figure 4:**
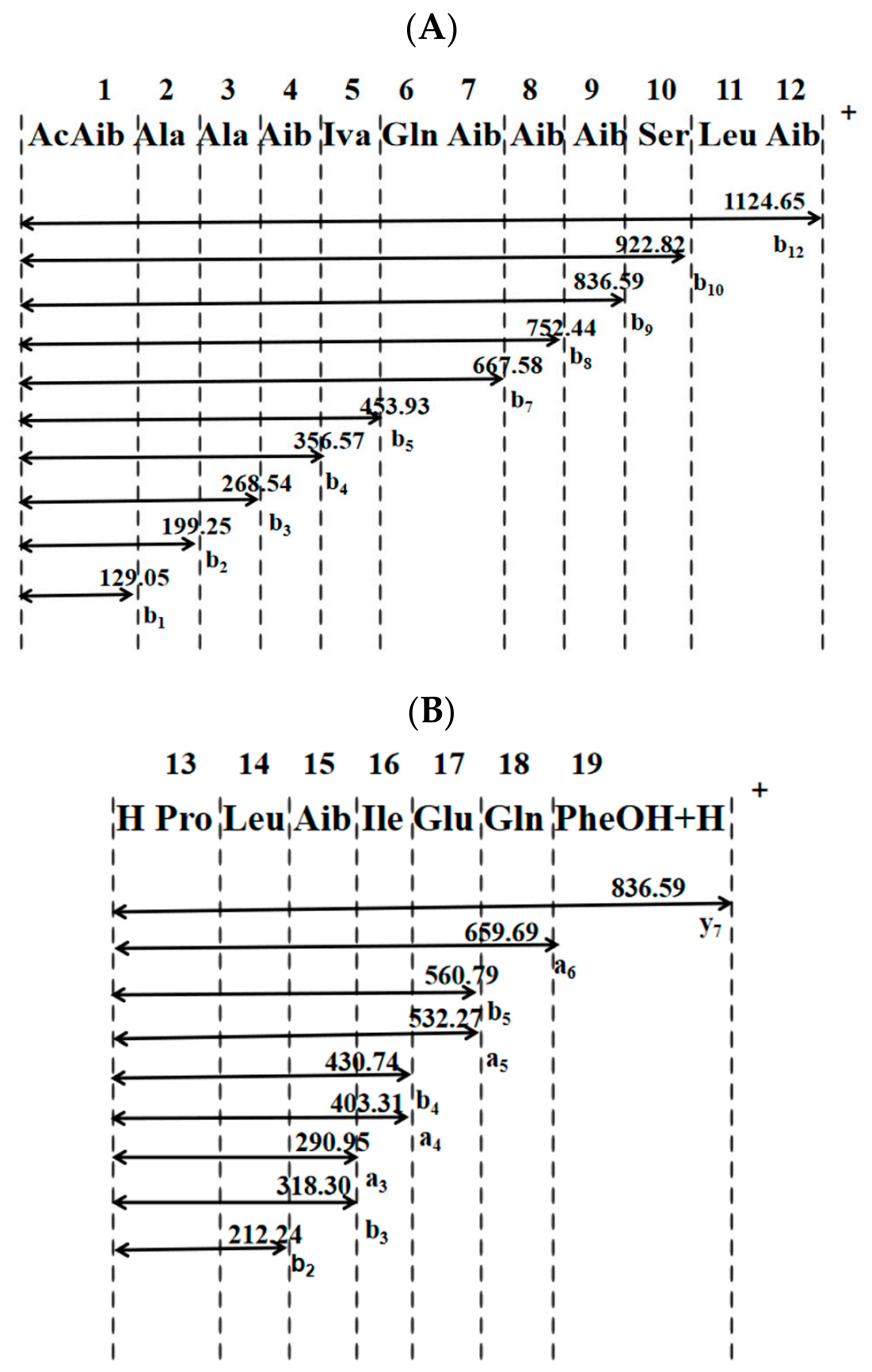
ESI-MS^2^ fragmentation pattern of trichorzianine 1938 (Compound **2**): (**A**) N-terminal fragment ion series; (**B**) complementary C-terminal fragment ion series, supporting the sequence assignment of Compound **2**.

Two complementary fragments (*m*/*z* 1124.65 and 836.59) from ESI-MS^2^ indicated cleavage between proline nitrogen and adjacent carbonyl groups, confirming the sequence: Ac-Aib^1^-Ala^2^-Ala^3^-Aib^4^-Iva^5^-Gln^6^-Aib^7^-Aib^8^-Aib^9^-Ser^10^-Leu^11^-Aib^12^-Pro^13^-Leu^14^-Aib^15^-Ile^16^-Glu^17^-Gln^18^-Pheol^19^ (Figure 3 and Figure 4).

This was consistent with the reported structure of trichorzianine 1938 [19].

### 2.3. Antibacterial Activity

#### 2.3.1. Previously Reported Bioactivities

Pentadecaibin IV has been reported to show cytotoxicity against human oral carcinoma KB cells (IC_50_ = 0.8 ± 0.2 μM) and acute toxicity to dipteran larvae (MED = 250 μg/mL), but no significant antibacterial activity against *S. aureus*, *E. coli*, or *Candida albicans* [20].

Trichorzianine 1938 displayed moderate antibacterial activity against several environmental bacterial strains (MIC = 12.5–200 μg/mL), but showed no activity against *E. coli*, suggesting species-specific antibacterial effects [19].

#### 2.3.2. Activity Against *Vibrio harveyi* VH306

**Figure 5:**
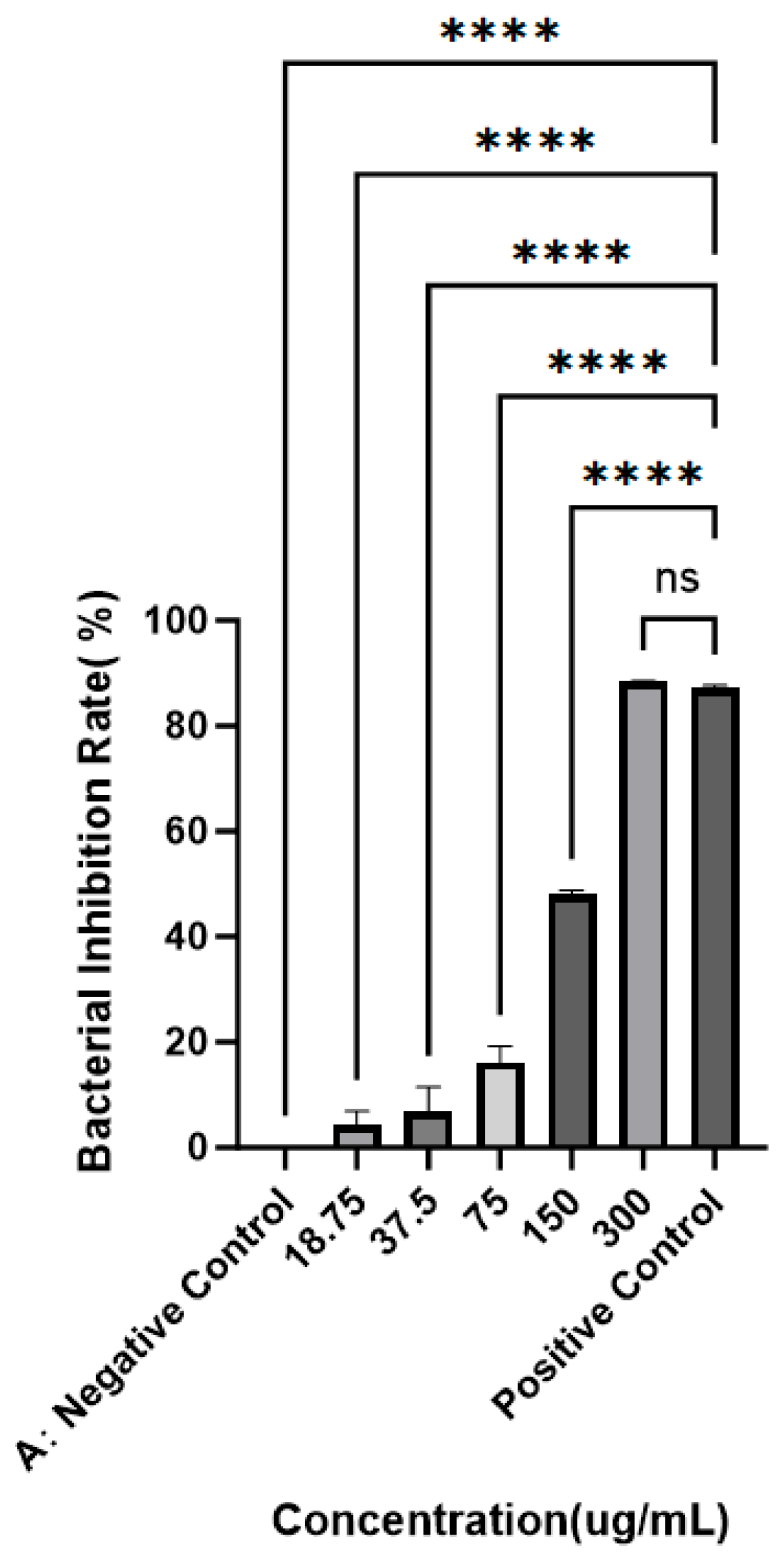
MIC determination of Compound **2** against *V. harveyi* VH306. A: Negative control served as baseline for inhibition rate calculation. **** *p* < 0.0001, ns: not significant.

In the present study, both compounds were tested against *V. harveyi* VH306. Trichorzianine 1938 (Compound **2**) exhibited measurable antibacterial activity with an MIC of 300 μg/mL (Figure 5), while pentadecaibin IV showed negligible inhibition. The results demonstrated a clear dose-dependent inhibition of bacterial growth by compound **2**. The inhibitory effect increased significantly with increasing concentrations, establishing a direct correlation between dosage and antibacterial efficacy.

The structural characteristics of peptaibols, such as N-terminal acetylation and C-terminal hydroxylation, facilitate their insertion into microbial membranes, forming transmembrane ion channels that lead to cell lysis [21]. Compared to Compound **1**, Compound **2** contained a longer amino acid sequence and a higher proportion of hydrophobic residues, such as proline and isoleucine. Hydrophobic enrichment has been reported to enhance peptide stability, accelerate bactericidal activity, and improve target recognition [22,23,24]. This structural feature may explain the superior antibacterial activity of trichorzianine 1938 (Compound **2**).

### 2.4. Molecular Docking Analysis

**Figure 6:**
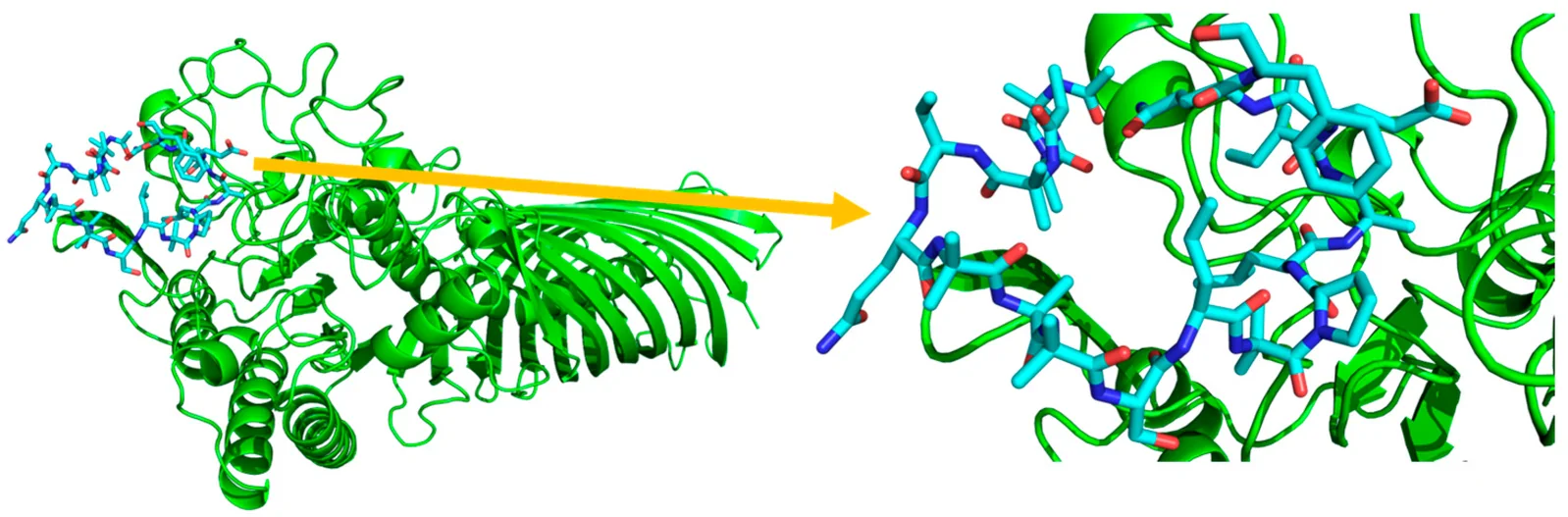
Binding interactions of Full-Length Autotransporter (3KVN) and Trichorzianine 1938. Orange arrow: transition from the overall view to a focused view of the chemical structure.

**Figure 7:**
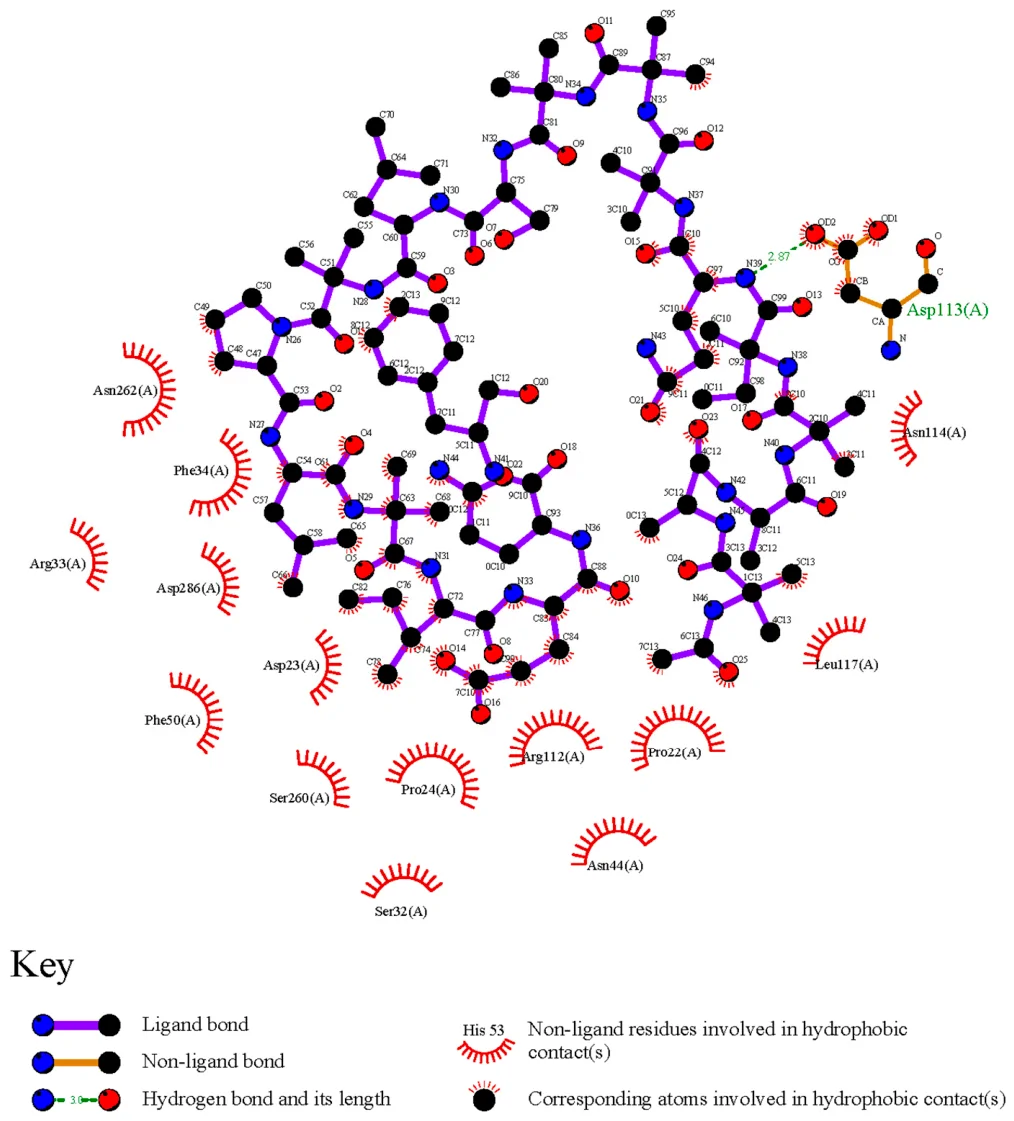
Two-dimensional interaction diagram of Full-Length Autotransporter (3KVN) in complex with Trichorzianine 1938 generated by LigPlot+.

As shown in Table 3, docking of Trichorzianine 1938 against the Full-Length Autotransporter (PDB ID: 3KVN) produced a LibDock score of 33.04 (Table 3). The corresponding binding interactions are presented in Figure 6, and the two-dimensional interaction diagram generated by LigPlot+ (V2.3.1) is shown in Figure 7. A comprehensive summary of the hydrogen bond and hydrophobic contacts is presented in Figure 7 and Table 4.

According to the LigPlot+ analysis (Figure 7 and Table 4), Trichorzianine 1938 forms a single hydrogen bond with ASP113(A) via side-chain atoms, serving as the primary polar anchor within the binding pocket. In addition, the ligand forms extensive hydrophobic and non-bonded interactions with multiple surrounding residues. The most prominent interactions were observed with PHE34(A) (17 contacts), ARG112(A) (13 contacts), PRO24(A) (13 contacts), and ASN262(A) (13 contacts), which appear to constitute the hydrophobic core of the binding site. Additional stabilizing interactions involve ASN44(A), PHE50(A), ASP23(A), PRO22(A), SER32(A), SER260(A), LEU117(A), ASN114(A), and ASP286(A), which collectively provide a complementary hydrophobic network surrounding the ligand. This distribution of polar and nonpolar interactions indicates that Trichorzianine 1938 is tightly accommodated within the active-site pocket, with ASP113(A) functioning as a critical anchoring residue, and the surrounding hydrophobic cluster reinforcing affinity and specificity.

## 3. Discussion

The purification results demonstrated that the fractions ST-27-4-3-1-3 (21.3 mg) and ST-27-4-3-1-6 (19.6 mg) were effectively isolated from the *Trichoderma longibrachiatum* ST-27 extract through a multi-step chromatographic process. Analytical HPLC under isocratic conditions, using a C18 reversed-phase column and a mobile phase of acetonitrile–water containing 0.1% trifluoroacetic acid, confirmed that both compounds were highly pure. The use of multi-wavelength detection at 210, 230, 254, 280, and 310 nm ensured that the assessment covered potential chromophoric regions associated with peptide-like metabolites (Figure 2 and Figure 4). The combination of gel column fractionation, silica gel chromatography, and semi-preparative HPLC provided efficient enrichment and purification, yielding samples of sufficient quantity and quality for subsequent structural elucidation. The results indicate that the isolation and purification procedures employed were appropriate and effective for obtaining pure monomeric compounds suitable for further spectroscopic characterization by ^1^H NMR, ^13^C NMR, and HRMS.

Spectroscopic and mass-spectrometric analyses identified both metabolites from *T. longibrachiatum* ST-27 as peptaibol-type peptides. Compound **1** displayed an [M + H]^+^ ion at *m*/*z* 1484.83 (C_70_H_117_N_17_O_18_) (Figure 2). The presence of Aib, Gln, Gly, Ala, Leu, and a terminal phenyl group in its NMR spectra, together with the MS^2^-derived sequence Ac-Aib^1^–Gly^2^–Ala^3^–Leu^4^–Aib^5^–Gln^6^–Iva^7^–Leu^8^–Aib^9^–Ala^10^–Aib^11^–Aib^12^–Aib^13^–Gln^14^–Pheol^15^, confirmed its correspondence to pentadecaibin IV (Table 1). 

Compound **2** gave an [M + H]^+^ ion at *m*/*z* 1939.11 (C_91_H_151_N_21_O_25_) (Figure 4) and showed multiple Aib residues typical of peptaibols. The MS^2^ fragments (*m*/*z* 1124.65 and 836.59) (Figure 4) and the deduced sequence Ac-Aib^1^–Ala^2^–Ala^3^–Aib^4^–Iva^5^–Gln^6^–Aib^7^–Aib^8^–Aib^9^–Ser^10^–Leu^11^–Aib^12^–Pro^13^–Leu^14^–Aib^15^–Ile^16^–Glu^17^–Gln^18^–Pheol^19^ matched trichorzianine 1938 (Table 2). Overall, the data verify that the isolated compounds correspond to pentadecaibin IV and trichorzianine 1938 (Figure 1 and Figure 3; Table 1 and Table 2).

The antibacterial evaluation showed that trichorzianine 1938 (Compound **2**) exhibited measurable inhibition against *Vibrio harveyi* VH306 with an MIC of 300 μg/mL (Figure 5), whereas pentadecaibin IV (Compound **1**) showed negligible activity. The inhibition by Compound **2** increased in a dose-dependent manner, indicating a positive correlation between concentration and bacterial growth suppression. Such a difference in efficacy can be attributed to the structural characteristics of peptaibols. Their N-terminal acetyl and C-terminal hydroxyl groups facilitate insertion into microbial membranes to form transmembrane ion channels, leading to cell lysis [21]. Compared to Compound **1**, Compound **2** possesses a longer peptide chain and a higher proportion of hydrophobic residues, including proline and isoleucine, which have been associated with enhanced peptide stability and membrane-binding affinity [22,23,24]. These features likely contribute to the stronger antibacterial activity observed for trichorzianine 1938. Although pentadecaibin IV and trichorzianine 1938 are known peptaibols, their biological origins and antibacterial profiles in the present study differ from those previously reported. Pentadecaibin IV was originally isolated from the marine sediment-derived *Trichoderma* sp. MMS1255 belonging to the *T. harzianum* species complex and was reported to exhibit cytotoxic activity but no appreciable antimicrobial activity against the tested bacterial and fungal strains [18]. Consistent with this previous observation, pentadecaibin IV showed negligible inhibition against *V. harveyi* VH306 in the present study. In contrast, trichorzianine 1938 was previously isolated from sponge-associated *T. atroviride* NF16 and showed antibacterial activity against several environmental bacterial isolates, with MIC values ranging from 12.5 to 200 μg/mL [19]. In the present study, trichorzianine 1938 isolated from the marine-derived *T. longibrachiatum* ST-27 exhibited measurable inhibition against *V. harveyi* VH306 with an MIC of 300 μg/mL. The comparatively higher MIC observed against *V. harveyi* suggests that the antibacterial susceptibility to trichorzianine 1938 may be species dependent. Therefore, the originality of the present study lies not in the discovery of new chemical structures, but in the different producing strain, the direct comparison of two structurally distinct known peptaibols, and their differential antibacterial activities against the aquaculture pathogen *V. harveyi* VH306.

Although the strain exhibited promising antibacterial activity under the current solid fermentation conditions, the fermentation parameters were not systematically optimized in this study. Factors such as salinity, carbon/nitrogen source composition, and fermentation duration may affect the production and accumulation of bioactive metabolites. Therefore, further optimization of fermentation conditions using systematic approaches, such as response surface methodology, is required to enhance metabolite yield and fully explore the biosynthetic potential of this strain.

In this study, the antibacterial activity was primarily evaluated based on OD_600_ measurements, which reflect bacterial growth inhibition but cannot completely distinguish bacteriostatic and bactericidal effects. Moreover, although the observed antibacterial activity suggested a possible membrane-related mechanism, direct evidence of membrane disruption was not obtained. Further investigations, including CFU counting, membrane permeability assays, and microscopic observations, are necessary to validate the antibacterial mechanism and provide deeper insights into the mode of action of the active metabolites.

As summarized in Table 3, docking of Trichorzianine 1938 against the Full-Length Autotransporter (3KVN) yielded a LibDock score of 33.04, and the top-ranked pose is shown in Figure 6. Figure 7 and Table 3 and Table 4 further indicate that the ligand forms a single hydrogen bond with ASP113(A) mediated via side-chain atoms. Although only one polar contact is observed, hydrogen bonds are among the most frequently occurring and functionally significant interactions in protein–ligand complexes, accounting for a substantial fraction of noncovalent contacts [25,26]. Such interactions are known to confer directional specificity and contribute markedly to binding affinity and selectivity [26]. Trichorzianine 1938 contains multiple peptide carbonyl and amide groups, together with polar functionalities contributed by Gln, Glu, Ser, and the terminal phenylalaninol residue. These structural features provide potential hydrogen bond donor and acceptor sites that may contribute to the predicted polar interaction with the receptor. However, the present docking analysis does not identify which specific functional group of Trichorzianine 1938 is responsible for the hydrogen bond with ASP113(A).

Beyond this polar anchor, the ligand is further stabilized by a network of hydrophobic (non-bonded) contacts. Notably, PHE34(A) (17 contacts), ARG112(A) (13 contacts), PRO24(A) (13 contacts), and ASN262(A) (13 contacts) stand out, indicating extensive non-bonded interactions around the predicted binding region. Hydrophobic contacts are the second-most common type of protein–ligand interactions (~28%) [27]. Such clusters likely contribute to shielding the ligand from solvent exposure and reinforcing binding through favorable van der Waals interactions. Trichorzianine 1938 contains several hydrophobic residues, including Aib, Iva, Leu, Ile, and Pro, together with the aromatic group of the terminal Pheol residue. These structural characteristics may contribute to the extensive non-bonded contacts observed in the docking model. Other residues, including ASN44(A), PHE50(A), ASP23(A), PRO22(A), SER32(A), SER260(A), LEU117(A), ASN114(A), and ASP286(A), also engage in stabilizing contacts, collectively surrounding the ligand in three dimensions. This interaction pattern is consistent with the well-established principle that effective ligand binding often relies on a combination of limited polar anchors and extensive hydrophobic contacts. [26,27]. Accordingly, the predicted interaction of Trichorzianine 1938 with the receptor appears to involve both polar recognition and hydrophobic stabilization, which is consistent with the coexistence of polar peptide groups and abundant hydrophobic residues in the peptaibol structure.

The binding mode is characterized by a single directional hydrogen bond, anchored through ASP113(A), and a dense hydrophobic shell formed by both aromatic and non-aromatic residues. This dual mode of interaction—polar anchoring plus nonpolar enclosure—emphasizes the cooperative nature of ligand recognition, with ASP113(A) acting as a predicted polar anchoring residue, while the hydrophobic cluster enhances overall affinity and stability. From a functional perspective, such combined polar and hydrophobic interactions could theoretically influence the local structural environment, accessibility, or stability of the Full-Length Autotransporter and thereby affect its associated function. However, the docking analysis in this study was intended as an exploratory computational assessment of one possible protein–ligand interaction rather than as a comprehensive investigation of the antibacterial mechanism. The present docking results do not demonstrate inhibition of transport or secretion, and the functional implications of the predicted interaction therefore remain theoretical. Moreover, the use of a single Full-Length Autotransporter target cannot account for the potentially multiple antibacterial mechanisms of trichorzianine 1938, particularly the membrane-disruptive effects commonly associated with peptaibols.

Previous studies provide a broader biological context for examining this type of interaction. Waters et al. reported that secretion involved in the virulence of *Vibrio harveyi* is regulated by quorum sensing and demonstrated conservation of several regulatory components involved in bacterial secretion [28]. Ganin et al. showed that a signaling molecule originating from *Vibrio* could perturb membrane processes and influence receptor organization and signaling in Gram negative bacteria [29]. More recently, Caudal et al. demonstrated that marine-derived metabolites can alter biofilm formation and quorum sensing pathways in *V. harveyi*, supporting the ability of extracellular marine metabolites to influence bacterial surface behavior and signaling processes [30]. Together, these findings provide biological context for exploring interactions between marine derived metabolites and bacterial surface proteins, although the specific functional consequence of the predicted interaction between Trichorzianine 1938 and the Full-Length Autotransporter requires further experimental validation.

The above structure–activity rule is consistent with prior studies on *T. longibrachiatum* peptaibols. BALÁZS et al. verified that peptide length and hydrophobic residues govern membrane penetration, which explains the distinct activity of the two compounds here in [31]. Caracciolo et al. further illustrated that hydrophobic modification can strengthen Gram-negative bacterial inhibition, providing a strategy to optimize Pentadecaibin IV [32].

*V. harveyi* readily forms biofilms to resist inhibition; Petit et al. noted marine metabolites suppress this pathogen via dual membrane and biofilm interference [33], so related tests can be supplemented in follow-up work. Marine Trichoderma are rich sources of peptaibols such as Hyporientalin A [34], yet few reports target aquatic *vibrios*. Our work fills this research blank.

## 4. Materials and Methods

### 4.1. Materials

#### 4.1.1. Experimental Strain

The marine-derived strain *Trichoderma longibrachiatum* ST-27 was preserved at the Laboratory of the Marine Biotechnology Innovation Center, Third Institute of Oceanography, Ministry of Natural Resources. The *Vibrio harveyi* strain VH306 was obtained from the Marine Pharmacy Research Center of Xiamen Medical College and the College of Fisheries, Jimei University.

#### 4.1.2. Culture Medium

PDB liquid medium: potato 200 g, glucose 20 g, distilled water 1000 mL, pH 5.2 ± 0.2. PDA solid medium: PDB medium 24 g, agar 12 g, distilled water 1000 mL, pH 5.2 ± 0.2. FS1 solid fermentation medium: rice 100 g, cornmeal 0.15 g, soybean peptone 0.45 g, sea salt 1.5 g, distilled water 120 mL. LB broth (sugar-free): tryptone 10 g, yeast extract 5 g, NaCl 5 g, distilled water 1000 mL, pH 7.0 ± 0.2.

#### 4.1.3. Instruments

The following instruments were utilized in this study: an analytical high-performance liquid chromatography (HPLC) system (Infinity 1260, Agilent, Santa Clara, CA, USA); a superconducting nuclear magnetic resonance (NMR) spectrometer (AV-600, Bruker, Rheinstetten, Germany); an ultra-high-performance mass spectrometer (QExactive, Thermo Fisher Scientific, Waltham, MA, USA); semi-preparative liquid chromatography systems (LC-6AD, Shimadzu, Kyoto, Japan and Quicksep-10D, Beijing Huideyi Technology Co., Ltd., Beijing, China); an automatic fraction collector (BS-100A, Shanghai Huxi Analytical Instrument Co., Ltd., Shanghai, China); an ultrasonic cleaner (DS-080S, Donson Intelligent Technology Co., Ltd., Suzhou, China); an analytical balance (BSA224S, Sartorius Scientific Instruments (Beijing), China); a rotary evaporator (E-10010N, Zhengzhou Great Wall Industry and Trade Co., Ltd., Zhengzhou, China); a nitrogen generator (Peak Scientific NM32LA, Peak Scientific, Scotland, UK, distributed by Shanghai Beke Gas Instrument Trade Co., Ltd., Shanghai, China); a high-pressure autoclave (Autoclave-G154D, ZEALWAY, Wilmington, DE, USA); and a calcium flux detection workstation (Flex-Station3, Molecular Devices, San Jose, CA, USA, distributed by Xiamen Baocheng Biotechnology Co., Ltd., Xiamen, China).

#### 4.1.4. Consumables and Reagents

Consumables included Petri dishes, pipette tips (200 μL and 1 mL), micropipettes, beakers (50, 100, and 200 mL), graduated cylinders (100 and 200 mL), sample vials (5 and 20 mL), HPLC vials (1.5 mL), rotary evaporation flasks (250 mL and 1 L), TLC plates (Si60F254, Merck KGaA, Darmstadt, Germany), 0.22 μm NY membranes, disposable syringes (1 mL), Sephadex LH-20 gel, chromatographic columns, analytical column (UltimateXB-C18, 4.6 × 250 mm, Welch Materials Co., Ltd., Shanghai, China), semi-preparative column (SP ODS-A C18, 10 × 250 mm, YMC Co., Ltd., Tokyo, Japan), 96-well microplates, and culture tubes (25 × 200 mm).

Reagents included formic acid, sulfuric acid, bismuth potassium iodide, ninhydrin, trifluoroacetic acid (Macklin, Shanghai, China), DMSO-*d*_6_ (Macklin), chromatographic-grade acetonitrile and methanol (CNW, Mainzm, Germany), ethanol, ethyl acetate, dichloromethane, and methanol (Xilong Scientific, Guangdong, China).

### 4.2. Fermentation of T. longibrachiatum ST-27

#### 4.2.1. Strain Activation and Large-Scale Fermentation

The method was adapted from Wu et al. [35] with modifications. PDA plates were sterilized by autoclaving (121 °C, 30 min), sealed with sterile film, and UV-irradiated for 30 min under aseptic conditions. Frozen stock cultures of ST-27 were inoculated onto PDA plates and incubated at 28 °C for 48–72 h. Mycelial plugs (6 mm diameter) were obtained using a sterile puncher and used as a solid inoculum.

For liquid seed culture, mycelial plugs were transferred into 250 mL flasks containing 50 mL PDB and shaken at 180 rpm, 28 °C for 48 h. For large-scale fermentation, 5% inoculum (10 mL seed culture) was added to 1 L flasks containing 200 mL FS1 medium. The cultures were incubated statically at 28 °C for 30–42 days, and growth status was monitored periodically.

#### 4.2.2. Extraction of Fermentation Products

At the end of fermentation, solid cultures were extracted with methanol for 72 h. The extracts were filtered under vacuum, and the filtrates were combined after three repeated extractions. Concentration under reduced pressure at 45 °C yielded a crude methanol extract, which was further subjected to ultrasonic-assisted extraction with dichloromethane. The combined organic phases were concentrated to afford the crude extract ST-27-4 (15.78 g).

### 4.3. Isolation of Fermentation Products

#### 4.3.1. Primary Isolation

TLC analysis of ST-27-4 was performed using dichloromethane–methanol (5:1, *v*/*v*) as the mobile phase. Bismuth potassium iodide staining revealed orange-red spots, suggesting nitrogen-containing compounds, while ninhydrin staining produced blue–violet spots, indicating amino acid derivatives.

The crude extract was subjected to Sephadex LH-20 gel chromatography with methanol as the eluent at a flow rate of one drop every 6 s. Fractions were collected every 30 min, monitored by TLC under UV (245 and 365 nm), and visualized with bismuth potassium iodide. Fractions with similar TLC profiles were pooled, yielding ten fractions (ST-27-4-1 to ST-27-4-10).

Each fraction was analyzed by HPLC using a Welch UltimateXB-C18 analytical column (4.6 × 250 mm). Elution was carried out with methanol and 0.1% formic acid aqueous solution under a gradient (5–100% B, 0–30 min; 100% B, 30–35 min; re-equilibration at 5% B, 36–45 min). Detection wavelengths were set at 210, 230, 254, 280, and 310 nm.

#### 4.3.2. Further Purification

Based on the HPLC analysis results, fractions ST-27-4-3, ST-27-4-4, ST-27-4-6, ST-27-4-7, and ST-27-4-8 (8.27 g) were combined and subjected to silica gel column chromatography. Samples were adsorbed onto diatomite and packed onto a silica gel column (200–300 mesh) with dichloromethane–methanol (7:1, *v*/*v*) as the mobile phase. Eluates were monitored by TLC (UV 245/365 nm and bismuth potassium iodide staining), and similar fractions were combined, yielding one major subfraction: ST-27-4-3-1 (3.78 g). Based on the subsequent chromatographic analysis, ST-27-4-3-1 was selected for further purification by semi-preparative HPLC, whereas ST-27-4-3-2 was not subjected to subsequent structural or bioactivity analyses.

ST-27-4-3-1 was further purified by semi-preparative HPLC using an SP ODS-A C18 column (10 × 250 mm, 5 μm). Elution was performed with acetonitrile–water containing 0.1% TFA at a flow rate of 1.5 mL/min, column temperature 25 °C, and detection at 210 and 230 nm. The elution program consisted of isocratic 60% B over 65 min. Finally, two fractions, ST-27-4-3-1-3 (Compounds **1**) and ST-27-4-3-1-6 (Compounds **2**), were collected. An additional fraction, ST-27-4-3-1-1, was examined by ^1^H NMR (Figure S7).

However, the remaining constituents were obtained in extremely low yields, which brought substantial difficulties to structural elucidation and precluded subsequent bioactivity evaluation. Therefore, only two major compounds were selected for further comprehensive analysis.

#### 4.3.3. Structural Identification of Isolated Compounds

After purification, the isolated compounds were dissolved in DMSO-*d*_6_ and analyzed by ^1^H and ^13^C NMR spectroscopy. Molecular formulas were determined by HR-ESI-MS, and peptide sequences were further examined by ESI-MS^2^ fragmentation. Structural assignments were established by integrating the NMR and mass-spectrometric data and comparing them with previously reported data for pentadecaibin IV and trichorzianine 1938 [18,19].

### 4.4. Antibacterial Activity Assay

Pentadecaibin IV (Compound **1**; ST-27-4-3-1-3) and trichorzianine 1938 (Compound **2**; ST-27-4-3-1-6) were dissolved in methanol at 3 mg/mL. Norfloxacin (10 mg/mL) was used as the positive control. Pentadecaibin IV (Compound **1**; ST-27-4-3-1-3) and trichorzianine 1938 (Compound **2**; ST-27-4-3-1-6) were dissolved in methanol at 3 mg/mL for antibacterial activity evaluation.

Experimental verification confirmed that pentadecaibin IV does not possess inhibitory activity; therefore, the corresponding figure is not presented.

MIC assays were performed against *V. harveyi* VH306 in 96-well plates. Bacterial inocula (1% *v*/*v*) were grown in LB broth and adjusted to OD_600_ before use. Wells 1–9 of row A received 16 μL of compound solution, well 10 received 16 μL norfloxacin, and well 11 received 16 μL methanol (negative control). Each well was adjusted to 160 μL with bacterial suspension. The final concentrations of the compounds in row A were 300 μg/mL, and the positive control was 100 μg/mL. Serial two-fold dilutions were performed down the plate, generating concentrations of 150, 75, 37.5, and 18.75 μg/mL in rows B–E. Plates were incubated at 32 °C for 24 h, and bacterial growth was measured at OD_600_ to determine MIC values.

### 4.5. Docking Simulation

#### 4.5.1. Ligand Preparation

Molecular docking was carried out in BIOVIA Discovery Studio 2021 using the LibDock algorithm to evaluate binding affinity and interaction patterns between protein targets and selected ligands. The docking ligands included Pentadecaibin IV and Trichorzianine 1938, which were obtained from the fermentation metabolites of the marine-derived fungus *Trichoderma longibrachiatum* strain ST-27. Structural information for each compound was retrieved in Structure Data File (SDF) format along with its PubChem Compound Identification (CID) numbers, as summarized in Table 5. Before docking, hydrogen atoms were added, three-dimensional conformations were generated, and the ligands were optimized and stored for subsequent computational analyses.

#### 4.5.2. Protein Preparation

The Full-Length Autotransporter (PDB ID: 3KVN) was selected as the docking receptor to evaluate the potential interactions of Pentadecaibin IV and Trichorzianine 1938 with a complete autotransporter structure comprising an extracellular passenger domain and a β-barrel translocator domain formed by 12 strands [36]. Previous studies have demonstrated the importance of virulence related secretion, cell surface signaling, biofilm formation, and quorum sensing in *Vibrio harveyi* and related bacterial systems [28,29,30]. These findings provided a biological basis for selecting the Full-Length Autotransporter as a representative receptor for the docking analysis. The corresponding protein information, including its name and PDB accession code, is summarized in Table 6. Before docking, the protein structure was processed using the default preparation protocol to resolve structural inconsistencies, and the resulting structure was exported in PDB format for further analysis.

#### 4.5.3. Molecular Docking

Following the preparation of both protein and ligands, docking simulations were conducted using the LibDock module in BIOVIA Discovery Studio 2021. The results, summarized in Table 3, report the LibDock scores obtained from the binding spheres, with the top-scoring pose for each ligand–protein complex retained for further evaluation. After docking, the predicted binding conformations were examined by generating ligand–protein interaction diagrams with LigPlot+ v.2.3 to visualize hydrogen bonds and hydrophobic contacts.

## 5. Conclusions

In this study, the marine-derived strain *Trichoderma longibrachiatum* ST-27 was systematically investigated for its secondary metabolites. Through a combination of silica gel chromatography, gel permeation chromatography, and semi-preparative HPLC, two monomeric compounds with relatively high purity were successfully isolated. Structural elucidation using NMR and HRMS techniques identified two linear peptaibol compounds, pentadecaibin IV and trichorzianine 1938. Antibacterial assays revealed that trichorzianine 1938 demonstrated measurable inhibitory activity against *Vibrio harveyi* VH306 (MIC = 300 μg/mL), whereas pentadecaibin IV showed negligible inhibition. Molecular docking suggested that trichorzianine 1938 may interact with the Full-Length Autotransporter (3KVN) with a LibDock score of 33.04, with a predicted hydrogen bond with ASP113(A) and extensive hydrophobic contacts that may contribute to stabilization of the predicted complex. These docking results indicate only a theoretical possibility of interaction and require further experimental validation. These findings provide further evidence for the antibacterial potential of peptaibol type metabolites from *T. longibrachiatum* ST-27. Overall, this study provides scientific evidence for the exploration of bioactive metabolites from *Marine fungi*. The results support further investigation of marine derived peptaibols as potential antibacterial candidates against aquaculture-associated pathogens; however, additional mechanistic and in vivo studies are required before their practical application can be established.

## Figures and Tables

**Table 1:** ^1^H and ^13^C NMR of pentadecaibin IV (1) (^1^H 500 MHz, ^13^C 126 MHz DMSO-*d*_6_).

Pos.	*δ*_C_	δ_H_, Mult. (*J* in Hz)	Pos.	*δ*_C_	*δ*_H_, Mult. (*J* in Hz)
Ac-Aib^1^			Gly^2^		
C=O	176.2	-	C=O	171.6	-
N−H	-	8.05,s	N−H	-	7.81,brs
*α* C	55.5	-	*α* CH_2_	44.8	3.81,dd(16.2, 6.61)
*β* CH_3_	26.4	1.49,s			3.63,dd(16.2, 5.01)
*β′* CH_3_	26.3	1.48,s			
COCH_3_	170.7	-			
COCH	23.1	1.93,s			
Ala^3^			Leu^4^		
C=O	175.2	-	C=O	173.3	-
N−H	-	7.85,d(5.93)	N−H	-	7.71,d(6.63)
α CH	51	3.97,dq(5.91, 7.51)	*α* CH	53.8	3.97,m
β CH_3_	16.8	1.36,d (7.55)	*β* CH_2_	36.6	1.75,m
				-	1.49,m
			*γ* CH	24.3	1.53,m
			*δ* CH_3_	22.1	0.83,d(6.32)
			*δ′* CH_3_	21.6	0.84,d(6.58)
Aib^5^			Gln^6^		
C=O	176.1	-	C=O	173.3	7.71, d(4.11)
N−H	-	8.05,s	N−H	-	3.73,m
*α* C	55.5	-	*α* CH	56.3	1.81,m
*β* CH_3_	26.0	1.47,s	*β* CH_2_	26.1	1.80,m
*β′*CH_3_	26.1	1.48,s		-	2.26,m
			*γ* CH_2_	31.5	2.12,m
			*δ* C=O *ε*	173.2	-
			NH_2_αn *ε*	-	7.20,s
			NH_2_sy	-	6.73,s
Iva^7^			Leu^8^		
C=O	171.4	-	C=O	172.9	-
N−H	-	7.81,brs	N−H	-	7.71, m
*α* C	43.9	-	*α* CH	54.7	3.78,m
*β* CH_2_	24.7	3.73,dd(16.2, 6.65)	*β* CH_2_	39.1	1.72,m
*β′* CH_3_	22.1	3.71,dd(16.2, 5.02)		-	1.53,m
*γ* CH_3_	7.6		*γ* CH	24.5	1.78,m
			*δ* CH_3_	21.6	0.83,d (6.53)
			*δ′* CH_3_	21.1	0.81,d (6.56)
Aib^9^			Ala^10^		
C=O	175.8	-	C=O	174.1	-
N−H	-	7.97,s	N−H	-	7.92, d (4.71)
*α* C	55.9	-	*α* CH	51.3	3.86, m
*β* CH_3_	25.8	1.43,s	*β* CH_3_	16.8	1.35, d (5.53)
*β′* CH_3_	25.9	1.48,s			
Aib^11^			Aib^12^		
C=O	175.2	-	C=O	171.4	-
N−H	-	7.86,s	N−H	-	7.81,brs
*α* C	55.5	-	*α* CH_2_	43.9	3.73,dd(16.2, 6.68)
*β* CH_3_	24.3	1.39,s		-	3.71,dd(16.2, 5.03)
*β′* CH_3_	22.7	1.42,s			
Aib^13^			Gln^14^		
C=O	174.1	-	C=O	171.2	-
N−H	-	7.99,s	N−H	-	7.38,m
*α* C	55.9	-	*α* CH	54.3	3.82,m
*β* CH_3_	22.1	1.36,s	*β* CH_2_	26.5	2.03,m
*β′* CH_3_	21.5	1.38,s		-	1.92,m
			*γ* CH_2_	31.5	2.17,m
				-	2.05,m
			*δ* C=O	173.5	-
			*ε* NH_2αnti_	-	6.97,s
			*ε* NH_2syn_	-	6.62s
Pheol^15^					
NH	-	7.11,m			
*α* CH	51.7	3.97,m			
*β* CH_2_	36.6	2.89,dd(13.6,4.42)			
	-	2.59,dd(13.6,9.10)			
*β′* CH_2_OH	73.1	3.31,m			
	-	3.38,m			
OH	-	4.53,dd(6.11,6.12)			
C-1	139.2	-			
C-2,6	129.2	6.24,m(d) (8.22)			
C-3,5	127.9	6.41,m(t) (8.38)			
C-4	126.2	6.20,m(t) (8.21)			

**Table 2:** 1H and 13C NMR of Trichorzianine 1938 (**2**) (1H 500 MHz, 13C 126 MHz DMSO-*d*_6_).

Pos.	*δ*_C_	δ_H_, Mult. (*J* in Hz)	Pos.	*δ*_C_	*δ*_H_, Mult. (*J* in Hz)
Ac-Aib^1^			Ala^2^		
C=O	175.6	-	C=O	174.1	-
N−H	-	8.61,s	N−H	-	8.25,d(5.21)
*α* C	55.8	-	*α* CH	51.3	4.06,m
*β* CH_3_	24.1	1.32,s	*β* CH_3_	16.5	1.32,d
*β′* CH_3_	26.1	1.35,s			
COCH_3_	171.1	-			
COCH	23.1	1.93,s			
Ala^3^			Aib^4^		
C=O	174.9	-	C=O	175.2	-
N−H	-	7.70,d(5.61)	N−H	-	7.88,s
*α* CH	51.3	3.99,m	*α* C	56.0	-
*β* CH_3_	16.2	1.33,d	*β* CH_3_	26.1	1.43,s
			*β′* CH_3_	23.1	1.35,s
Iva^5^			Gln^6^		
C=O	176.4	-	C=O	173.6	-
N−H	-	7.41,s	N−H	-	7.74,m
*α* C	58.8	-	*α* CH	56.0	3.79,m
*β* CH_2_	25.6	2.21,m	*β* CH_2_	26.3	1.97,m
		1.62,m	*γ* CH_2_	-	2.16,m
*β′* CH_3_	22.7	1.35,s		-	2.23,m
*γ* CH_3_	7.1	0.74,t	*δ* C=O	31.7	-
			*ε* NH_2αnti_	-	7.17,s
			*ε* NH_2syn_	173.3	6.76s
Aib^7^			Aib^8^		
C=O	175.8	-	C=O	176.0	-
N−H	-	7.86,s	N−H	-	7.54,s
*α* C	56.1	-	*α* C	56.1	-
*β* CH_3_	26.3	1.43,s	*β* CH_3_	26.3	1.35,s
*β′* CH_3_	23.1	1.33,s	*β′* CH_3_	23.1	1.38,s
Aib^9^			Ser^10^		
C=O	176.4	-	C=O	170.7	-
N−H	-	7.75,s	N−H	-	7.74,m
*α* C	56.1	-	*α* CH	58.9	4.06,m
*β* CH_3_	26.5	1.46,s	*β* CH_2_	61.9	3.68,m
*β′* CH_3_	23.1	1.42,s		-	3.79,m
			OH	-	-
Aib^11^			Aib^12^		
C=O	173.6	-	C=O	172.8	-
N−H	-	7.59,d(8.01)	N−H	-	7.91,s
*α* CH	51.3	4.25,m	*α* C	56.3	-
*β* CH_2_	39.7	1.62,m	*β* CH_3_	25.8	1.38,s
	-	1.71,m	*β′* CH_3_	23.1	1.49,s
*γ* CH	23.9	1.76,m			
*δ* CH_3_	23.1	0.72,d			
*δ′* CH_3_	19.7	0.83,d			
Aib^13^			Gln^14^		
C=O	173.4	4.23,t(8.11)	C=O	173.5	-
*α* CH	63.2	1.62,m	N−H	-	7.71,m
*β* CH_2_	28.6	2.23,m	*α* CH	53.8	3.91,m
	-	1.86,m	*β* CH_2_	37.9	1.51,m
*γ* CH_2_	26.3	3.48,m		-	1.76,m
*δ* CH_3_	48.2	3.68,m	*γ* CH	25.1	1.68,m
			*δ* CH_3_	23.1	0.83,d(6.32)
			*δ′* CH_3_	19.7	0.93,m
Pheol^15^			Ile^16^		
C=O	175.5	-	C=O	172.1	-
N−H	-	7.60,s	N−H	-	6.92,s
*α* C	56.3	-	*α* CH	58.9	3.93,m
*β* CH_3_	26.1	1.44,s	*β* CH	35.6	1.88,m
*β′* CH_3_	23.4	1.37,s	*γ* CH_3_	25.1	1.20,m
			*γ′* CH_2_	11.5	1.47,m
			*δ* CH_3_	15.6	0.82,m

**Table 3:** LibDock scores for the two compounds against Full-Length Autotransporter (3KVN).

Compounds	LibDock Score
Pentadecaibins IV	-
Trichorzianine 1938	33.04

- No binding.

**Table 4:** Interaction summary between the ligand and protein (from LigPlot+ analysis).

Interaction Type	Protein Residue (Chain A)	Details
Hydrogen bond	ASP113	One hydrogen bond involving side-chain atoms (S–S type)
Hydrophobic/non-bonded contacts	ASP286	1 contact
	ASN262	13 contacts
	SER260	1 contact
	LEU117	2 contacts
	ASN114	2 contacts
	ARG112	13 contacts
	PHE50	4 contacts
	ASN44	9 contacts
	PHE34	17 contacts
	ARG33	1 contact
	SER32	2 contacts
	PRO24	13 contacts
	ASP23	4 contacts
	PRO22	4 contacts

**Table 5:** Summary of compounds used as ligands.

Compounds	PubChem CID
Pentadecaibins IV	164618726
Trichorzianine 1938	163114856

CID, compound identification.

**Table 6:** Protein Target and Binding Sphere Coordinates.

Name	PDB ID	Binding Spheres (x, y, z)	Reference
Full-Length Autotransporter	3KVN	−10.24, 36.36, 94.75	[36]

## Data Availability

The original contributions presented in this study are included in the article/Supplementary Materials. Further inquiries can be directed to the corresponding author.

## Supplementary Materials

The following supporting information can be downloaded at https://www.mdpi.com/article/10.3390/ijms27177714/s1.

## Abbreviations

The following abbreviations are used in this manuscript:

CID	Compound ID
DMSO-*d*_6_	Deuterated Dimethyl Sulfoxide
ESI-MS^2^	Electrospray Ionization Tandem MS
FS1	Formula Solid medium 1
HPLC	High-Performance Liquid Chromatography
HRMS	High-Resolution Mass Spectrometry
HR-ESI-MS	High-Resolution (Electrospray Ionization) Mass Spectrometry
IC_50_	Half-Maximal Inhibitory Concentration
LB	Luria–Bertani broth
MED	Minimum Effective Dose
MIC	Minimum Inhibitory Concentration
NMR	Nuclear Magnetic Resonance
PDA	Potato Dextrose Agar
PDB	Protein Data Bank
SDF	Structure Data File
TFA	Trifluoroacetic Acid
TLC	Thin-Layer Chromatography
UV	Ultraviolet
VH306	*Vibrio harveyi* VH306
