# Supplementary material for: Activity of Secondary Metabolites from Marine-Derived Trichoderma longibrachiatum ST-27 Against Vibrio harveyi
Source: Int J Mol Sci. 2026 Aug 28;27(17):7714. doi: 10.3390/ijms27177714 (PMC13566436; doi:10.3390/ijms27177714)
Supplement: Supplementary file 1 [file ijms-27-07714-s001.zip › ijms-4425886-supplementary.pdf]

Supplementary Information

Contents

Figure S1. ^{1}H NMR of ST-27-4-3-1-3 ,500 MHz, DMSO-*d6*

Figure S2. ^{13}C NMR of ST-27-4-3-1-3, 126 MHz, DMSO-*d6*

Figure S3. HR-ESI-MS of ST-27-4-3-1-3

Figure S4.^{1}H NMR of ST-27-4-3-1-6 ,500 MHz, DMSO-d_6

Figure S5. ^{13}C NMR of ST-27-4-3-1-6, 126 MHz, DMSO-d_6

Figure S6. HR-ESI-MS of ST-27-4-3-1-6

Figure S7. ^{1}H NMR of **ST-4-3-1-1**,500 MHz, DMSO-d_6

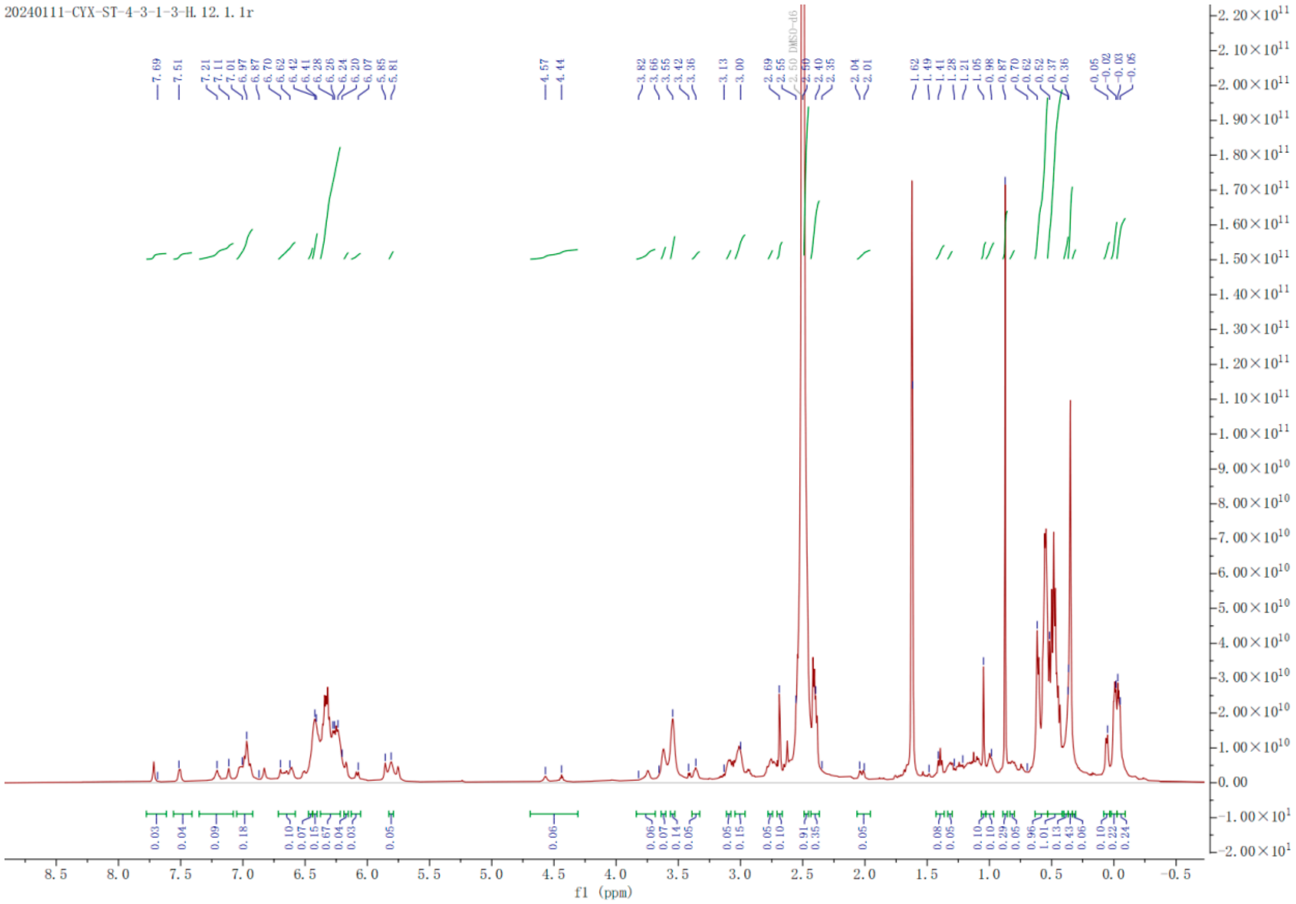

Figure S1. ^{1}H NMR of **ST-27-4-3-1-3** ,500 MHz, DMSO-*d6*

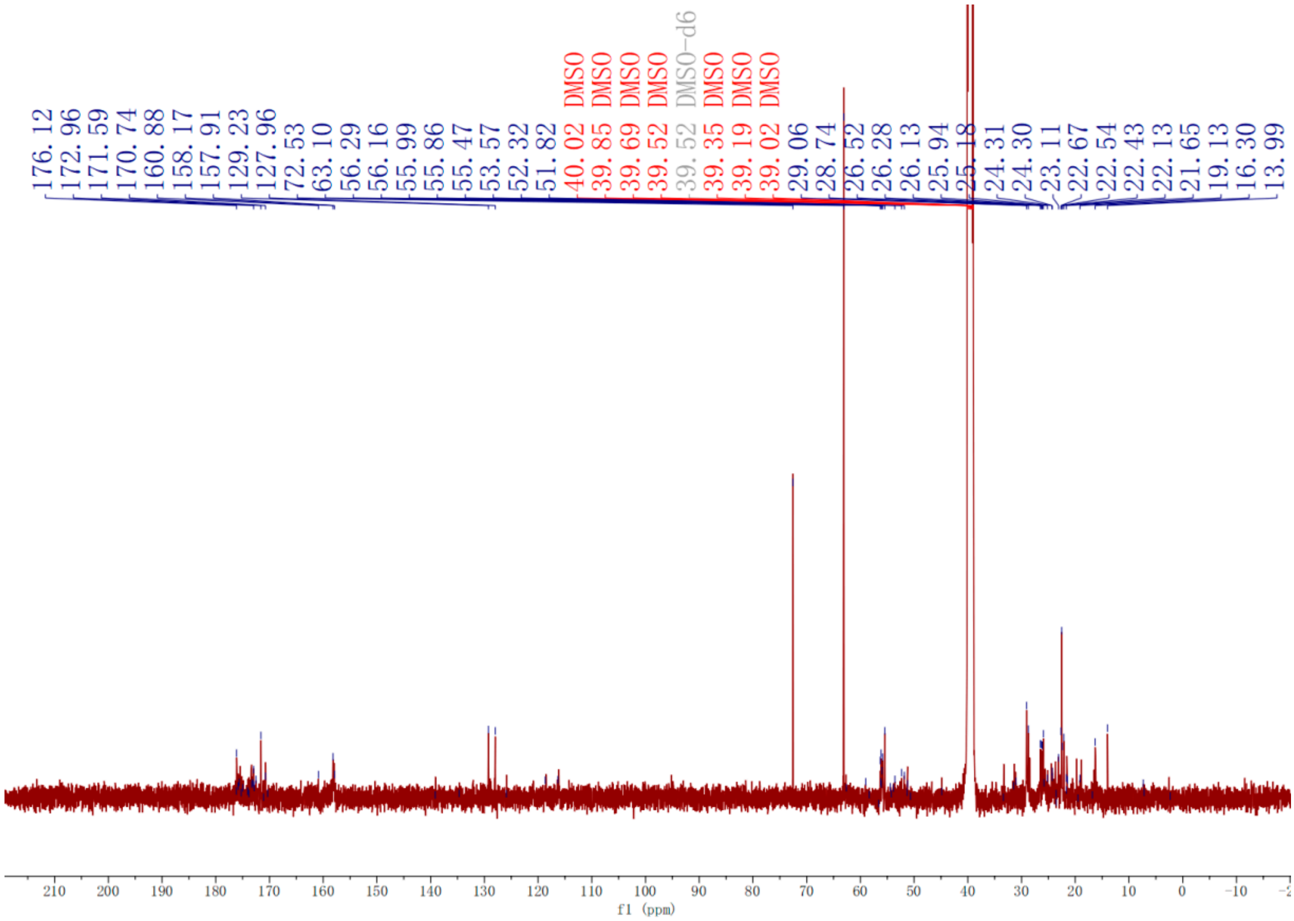

Figure S2. ^{13}C NMR of **ST-27-4-3-1-3**, 126 MHz, DMSO-*d6*

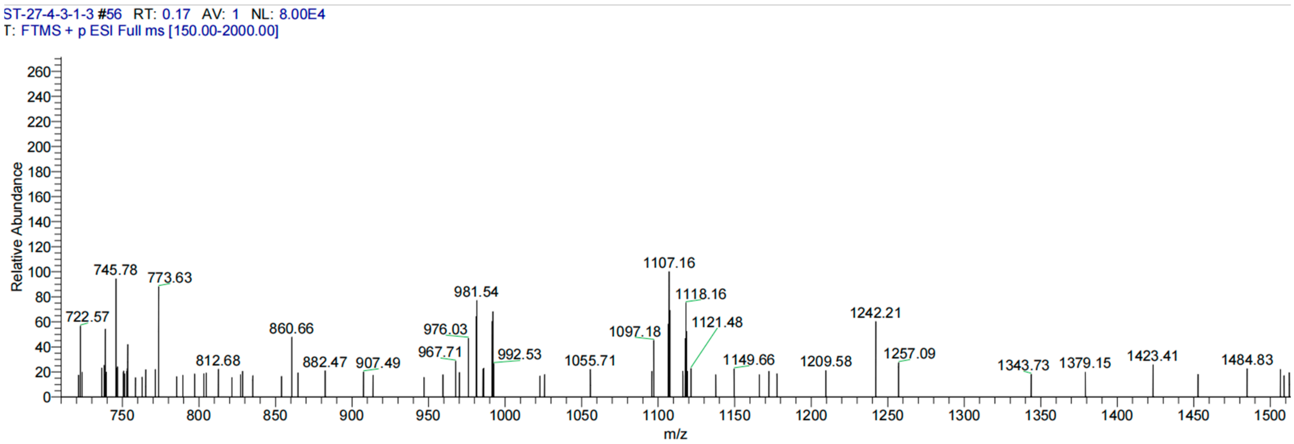

Figure S3. HR-ESI-MS of **ST-27-4-3-1-3**

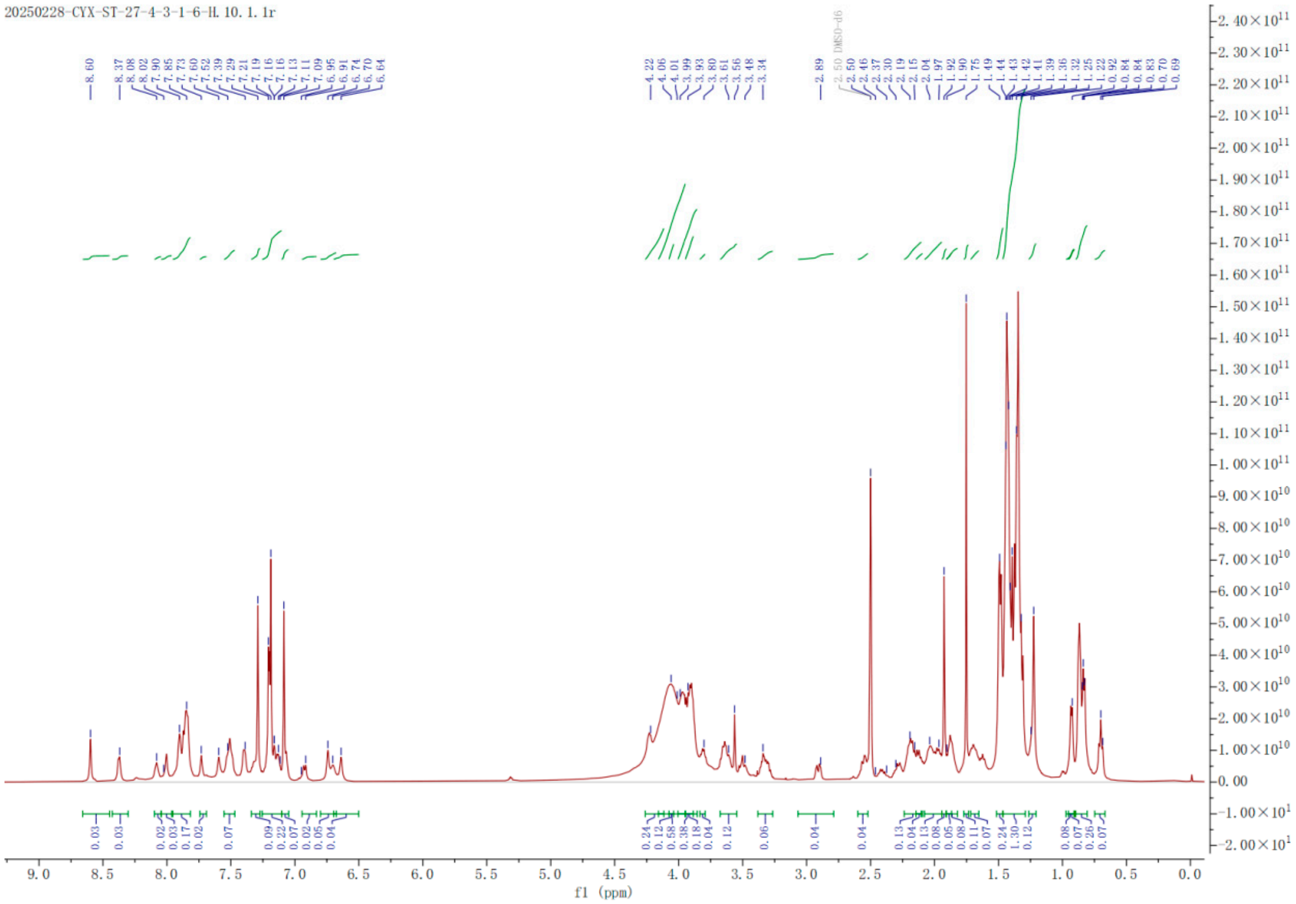

Figure S4.^{1}H NMR of **ST-27-4-3-1-6** ,500 MHz, DMSO-*d6*

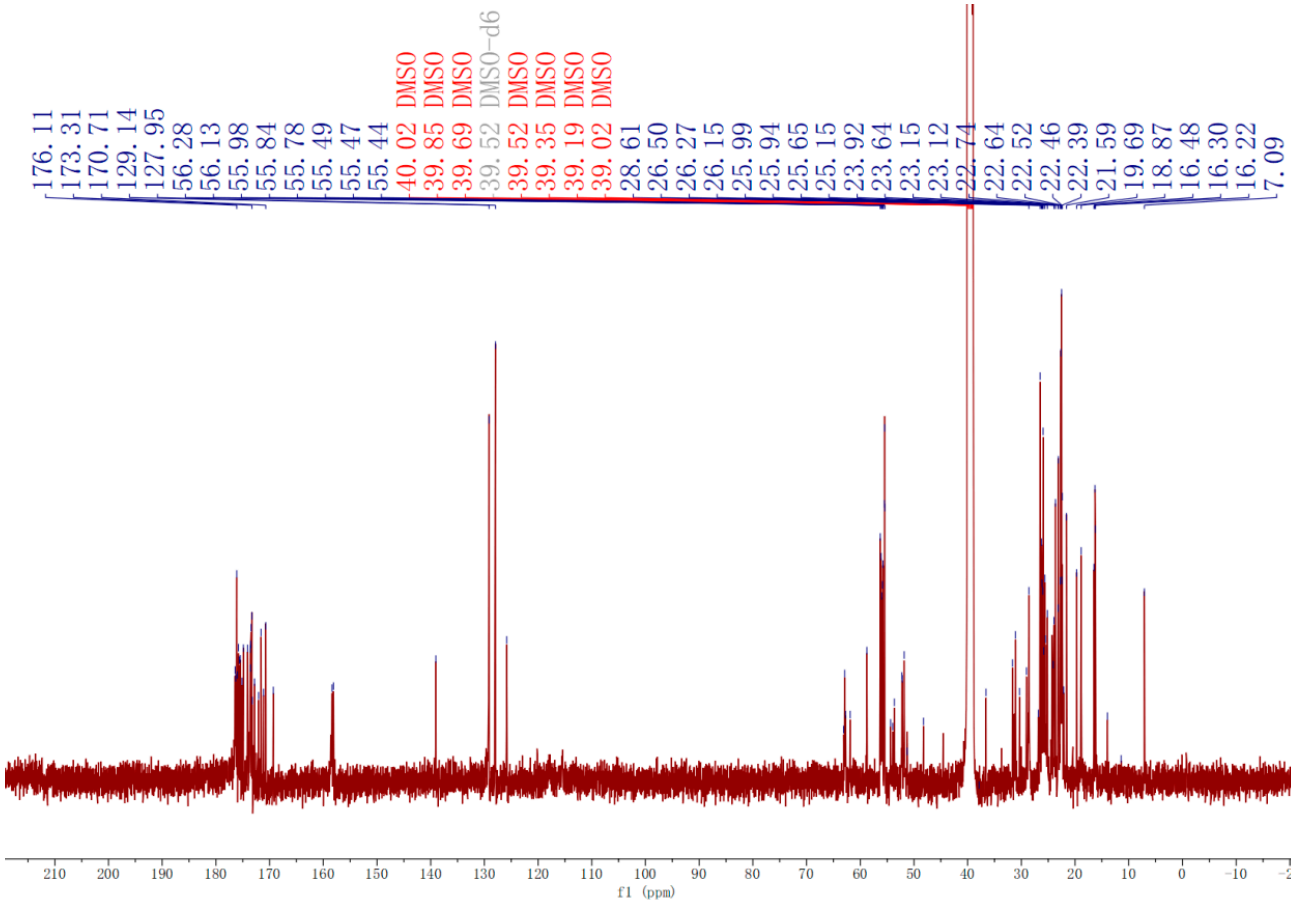

Figure S5. ^{13}C NMR of **ST-27-4-3-1-6**, 126 MHz, DMSO-d_6

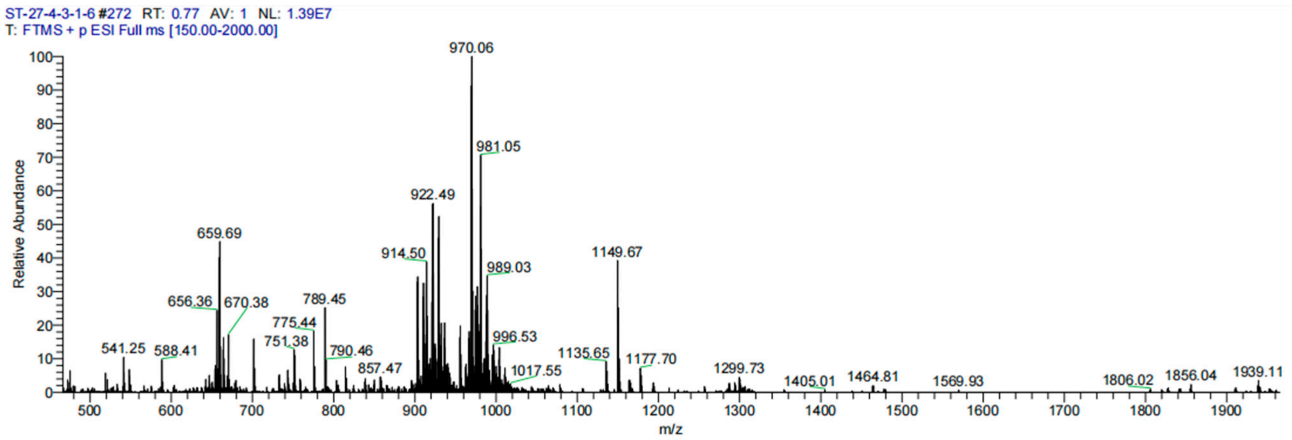

Figure S6. HR-ESI-MS of **ST-27-4-3-1-6**

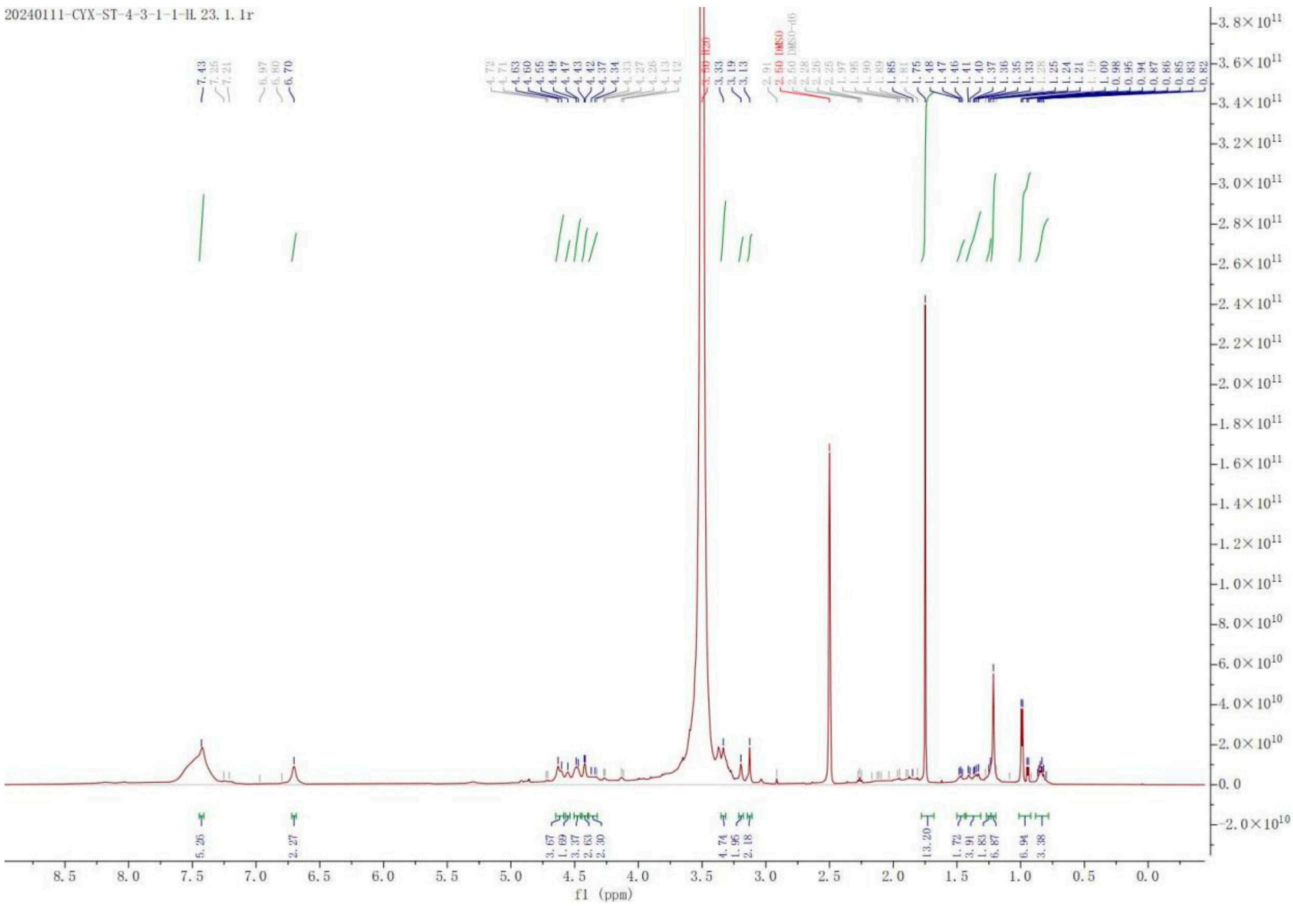

Figure S7. ^{1}H NMR of **ST-4-3-1-1**,500 MHz, DMSO-*d*$_6$
